# The role of mammalian Sirtuin 6 in cardiovascular diseases and diabetes mellitus

**DOI:** 10.3389/fphys.2023.1207133

**Published:** 2023-07-11

**Authors:** Kehan Wu, Yaqiao Wang, Runmin Liu, Hao Wang, Tao Rui

**Affiliations:** Division of Cardiology, Department of Medicine, The Affiliated People’s Hospital of Jiangsu University, Zhenjiang, Jiangsu, China

**Keywords:** Sirt6, molecular mechanisms, cardiovascular diseases, diabetes mellitus, biological function

## Abstract

Cardiovascular diseases are severe diseases posing threat to human health because of their high morbidity and mortality worldwide. The incidence of diabetes mellitus is also increasing rapidly. Various signaling molecules are involved in the pathogenesis of cardiovascular diseases and diabetes. Sirtuin 6 (Sirt6), which is a class III histone deacetylase, has attracted numerous attentions since its discovery. Sirt6 enjoys a unique structure, important biological functions, and is involved in multiple cellular processes such as stress response, mitochondrial biogenesis, transcription, insulin resistance, inflammatory response, chromatin silencing, and apoptosis. Sirt6 also plays significant roles in regulating several cardiovascular diseases including atherosclerosis, coronary heart disease, as well as cardiac remodeling, bringing Sirt6 into the focus of clinical interests. In this review, we examine the recent advances in understanding the mechanistic working through which Sirt6 alters the course of lethal cardiovascular diseases and diabetes mellitus.

## 1 Introduction

Cardiovascular diseases (CVDs) have been reported to be among the leading causes of human death worldwide (D'Onofrio et al., 2015). CVDs, which include coronary heart disease, heart failure, as well as hypertension, etc., claimed more lives than that of cancer and chronic obstructive pulmonary diseases combined (Li et al., 2021). This encouraged us to investigate the pathogenesis of CVDs and develop effective therapeutic approaches. It is known that aberrant biological processes in CVDs include mitochondrial dysfunction, apoptosis, and abnormal energy metabolism whereas risk factors contributing to the onset of cardiovascular diseases comprise of diabetes, inadequate calorie restriction, and obesity (Saiyang et al., 2021). Diabetes mellitus (DM) is also a notorious disease in clinical settings (Beckman et al., 2013) and even worsens clinical outcomes of patients with CVDs, especially patients with acute myocardial infarction (Goodarzi and Rotter, 2020). Therefore, it is imperative to understand the underlying mechanistic workings of CVDs and DM so that experimental discoveries can be translated into clinical treatments of these life-threatening maladies.

The Sirtuin protein family, which is composed of several highly conserved histone deacetylases that catalyze deacetylation of both histone and non-histone lysine residue, beneficially regulates lifespan and cell senescence (Imai et al., 2000). Unlike other classes of histone deacetylases, Sirtuins require nicotinamide adenine diphosphate (NAD+) for their enzymatic activities (Matsushima and Sadoshima, 2015). In addition to deacetylation activity, some of them possess other enzymatic activities such as demalonylation, desuccinylation, mono-ADP-ribosylation and glutarylation activities (Winnik et al., 2015). Sir2 (silent information regulator 2), which is also known as the founding member of Sirtuin family, was firstly discovered and described in the study of *Saccharomyces cerevisiae* (Saiyang et al., 2021). Mammals hold seven mammalian orthologs of Sir2, from Sirt1 to Sirt7, all possessing highly conserved catalytic domains and NAD + binding sites. These proteins were found in different cellular compartments respectively to exert a variety of physiological functions. Among them, Sirt1 is localized both in the nucleus and cytosol; Sirt2 is typically discovered in cytoplasm but is also found in nuclei in certain phase of cell cycle; Sirt3, Sirt4, and Sirt5 are exclusively located in mitochondria and exerts important effects on oxidative stress, together with lipid metabolism (Gimbrone and García-Cardeña, 2016); Sirt6 and Sirt7 are nuclear and nucleolar, respectively, with important regulatory roles in cellular processes such as gene expression and DNA repair (Tian et al., 2019). Notably, Sirt6 trans-locates to cytoplasm to interact with cytoplasmic stress granules upon stress, which means Sirt6 is not a protein that merely resides in the nucleus (Jedrusik-Bode et al., 2013).

Among Sirtuin family, Sirt6 has received tremendous attention because of unique enzymatic activities. Sirt6 has deacetylation activities and could target specific sites on histone 3 lysine 9 (H3K9) and histone 3 lysine 56 (H3K56). The deacetylation effect of Sirt6 is crucial for chromatin compaction, as well as transcriptional repression. Enzymatic activities of Sirt6 closely impacts its biological functions such as anti-inflammatory response effect and DNA damage repair (Tasselli et al., 2017). Studies have unveiled that compromised Sirt6 enzymatic activities, as well as its biological functions, result in the onset and development of many human diseases, including CVDs, metabolic diseases, and shortened lifespan (Vitiello et al., 2017). For example, Sirt6 deficient mice developed different types of diseases and symptoms such as diabetes, osteoporosis (Wang et al., 2021), cancer, curved spines, lymphopenia and decreased subcutaneous fat, resembling a progeroid-like syndrome (Sundaresan et al., 2012; Tan et al., 2021). Sirt6 is also a longevity protein whose overexpression in mice markedly increased lifespan compared with that of wild-type mice (Zhong et al., 2010). Interestingly, the heart has the highest Sirt6 protein expression levels in the body (Pereira et al., 2012), indicating that Sirt6 is most likely to regulate the pathophysiology of CVDs. Mounting evidence elaborating the roles of Sirt6 in cardiac disorders has emerged in recent years. For instance, Sirt6 depletion translocated FoxO1 to the heart, upregulating PDK4, reducing oxygen consumption and ATP production, thereby demonstrating a protective role of Sirt6 in maintaining cardiac homeostasis (Zhong et al., 2010). Therefore, extensive knowledge concerning the regulatory mechanisms of Sirt6 lays foundation for developing effective therapeutic approaches for lethal CVDs.

In this review, we first focus on the structure and biological functions of Sirt6 and then we seek to interrogate recent advances in our knowledge of roles of Sirt6 in CVDs and DM, with emphasis on the molecular mechanisms underlying its protective roles. We primarily examine the roles of Sirt6 in atherosclerosis, coronary heart disease, cardiac ischemia/reperfusion (I/R)-induced injury, diabetic cardiomyopathy, hypertension, cardiac hypertrophy, heart failure, cardiac fibrosis, and DM.

## 2 The structural basis of Sirt6

The mammalian Sirt6 comprises an NAD^+^-binding Rossmann fold domain and a zinc-binding domain, with additional N- and C- termini that direct cellular localization of Sirt6.

### 2.1 Rossmann fold domain and the binding ligand NAD^+^

Sirt6 has a Rossmann fold structural motif, which spans residues 27–132 and 195–268 and is architecturally featured by a six-stranded parallel β-sheet in between two helices on one side and four helices on the other (Pan et al., 2011), provides a platform for the NAD^+^ binding site. Adenosine diphosphate ribose (ADPr), the non-catalytic NAD^+^ surrogate deprived of the nicotinamide moiety, is often used in structural research to prevent catalytic turnover. Like NAD^+^, ADPr also occupies the NAD^+^ binding site. Analysis of bound ADPr unveiled 18 possible polar contacts between the Rossmann domain and ADPr. Also, many studies showed that mutations in NAD^+^ binding site leads to loss of deacetylase activity of Sirt6 in human cancer cells (Kugel et al., 2015), human fibroblasts (McCord et al., 2009), and 293T cells (Klein et al., 2020), indicating that the NAD^+^ binding site in the Rossmann domain is responsible for the deacetylase activity of Sirt6. Since most signaling transduction and biological functions mediated by Sirt6 are attributed to its deacetylase activity (Tennen and Chua, 2011; Etchegaray et al., 2013; Jedrusik-Bode et al., 2013; Kugel and Mostoslavsky, 2014; Winnik et al., 2015), Sirt6 interacts with multiple signaling transduction and phosphorylation through the Rossmann domain. Also, through binding with NAD^+^, there is an interplay between Sirt6 and energy metabolic signal cascades.

### 2.2 Zinc-binding domain

The Sirt6 zinc domain has four cysteine residues to coordinate Zn^2+^, and Sirt6 incorporates a 10-amino acid insert between the third and fourth cysteine (Pan et al., 2011). It is reported that loss of activity was due to repositioning of the Zn^2+^-binding domain following zinc release, disrupting NAD^+^ binding (Wood et al., 2018).

### 2.3 N- and C- termini

It is discovered that the N terminus of Sirt6 is crucial for H3K9 and H3K56 deacetylase activity and for chromatin association (Tennen et al., 2010). Also, N terminus of Sirt6 can also be phosphorylated by c-JUN N- terminus kinase (JNK), recruiting PARP1 to DNA damage sites to promote restoration (Van Meter et al., 2016). Miteva and Cristea, 2014 found that phosphorylation of the C- terminus may regulate protein-protein interactions, however, no additional evidence suggested the cellular function for this interaction.

## 3 The biological functions of Sirt6

**FIGURE 1:**
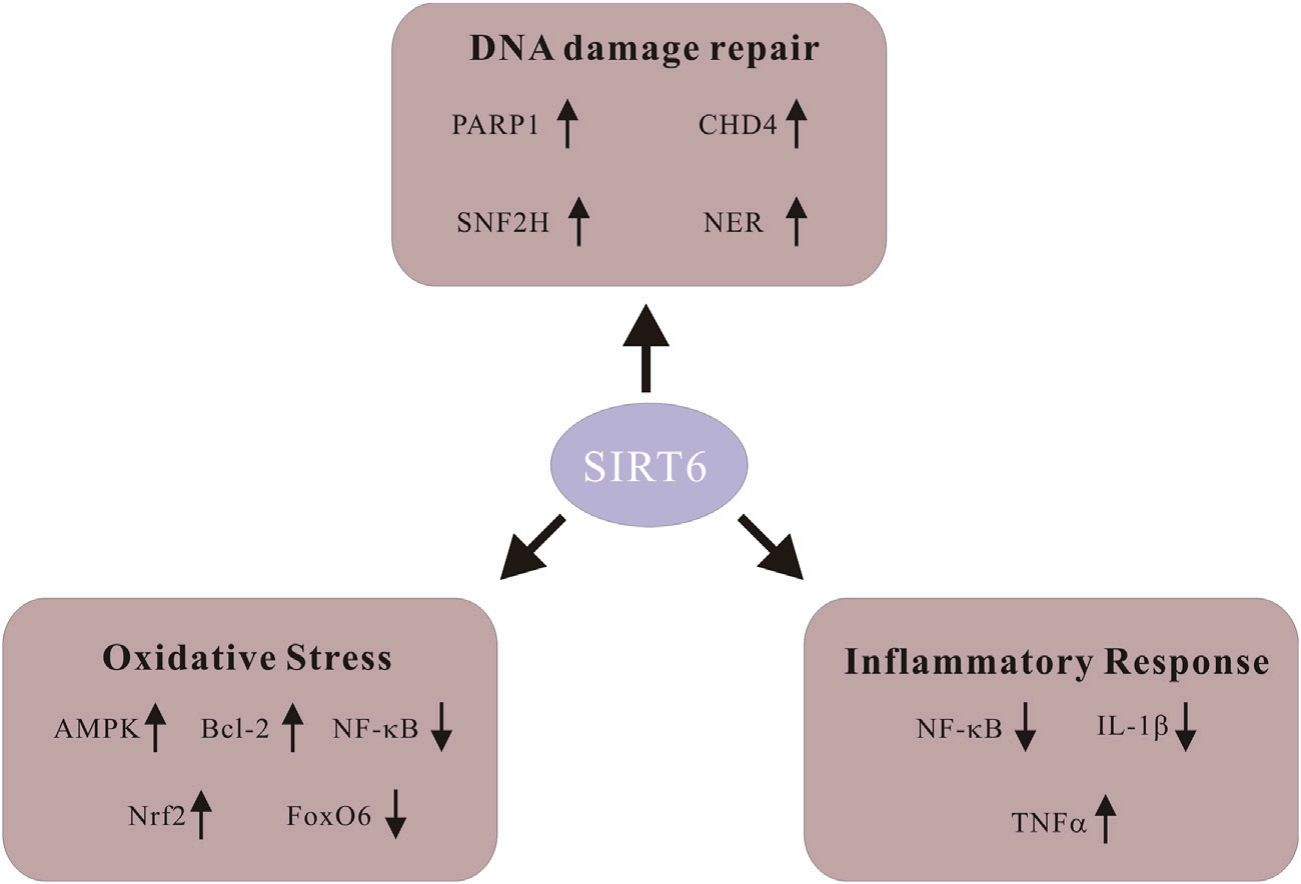
Schematic of signaling molecules in various biological functions of Sirt6. Sirt6 activates poly-ADP-ribose polymerase 1 (PARP1) and promotes DNA damage repair. Sirt6 restores DNA damage through global genome nucleotide excision repair (NER). Sirt6 recruits SNF2H, a chromatin remodeler whose function is to safeguard against genomic instability. Sirt6 coordinates with chromodomain helicase DNA-binding protein 4 (CHD4) to repair DNA damage. Sirt6 activates AMPK signaling, upregulates Bcl-2 and inhibits NF-KB to alleviate ROS level. Sirt6 enhances Nrf2 abundance and exhibits antioxidant activity. Downregulation of FoxO6 upregulates Sirt6 and alleviates cardiomyocyte oxidative stress. Sirt6 represses NF-KB activity through various means and decreases production of the pro-inflammatory cytokine IL-1β. Sirt6 expedites the generation as well as releasing of inflammatory cytokine TNF-α.

Among its numerous biological functions, the contribution of Sirt6 to DNA damage repair puts emphasis on its significant potential as a promising therapeutic target for aging-related diseases (Winnik et al., 2015). Moreover, oxidative stress and inflammatory response are also crucial in the progression of CVDs, and Sirt6 is also involved in it (Pereira et al., 2012). Since the Sirt6 exerts its biological effects on the regulation of various diseases, it is pivotal to review them in detail (Figure 1).

### 3.1 Sirt6 promotes DNA damage repair

The fundamental role of Sirt6 in restoring DNA damage was initially postulated by researchers claiming that mouse cells with Sirt6 depletion exhibit increased sensitivity to DNA-damage agents, stalled proliferation, genomic instability and a progeroid syndrome (Tan et al., 2021). Besides, a paper reported that Sirt6 repairs oxidative stress-induced DNA damage by activating PARP1 (Mao et al., 2011). In addition, Sirt6 promotes DNA double strand break (DSB) repair by stabilizing DNA-dependent protein kinase (McCord et al., 2009). These were found at the early stage of the discovery of Sirt6.

However, with the advance of scientific research, more evidence elaborating the interplay between Sirt6 and DNA damage repair has been presented. For example, the efficiency of restoring DNA DSB is proportional to maximum lifespan; it is known that Sirt6 is associated with cell senescence; therefore, it is logical to postulate a hypothesis that Sirt6 may accelerate the rate at which DNA DSB damage is repairing. Fortunately, this has been confirmed by several studies claiming that Sirt6 is indeed capable of stimulating DNA DSB repair in long-lived species (Tian et al., 2019). Mechanistically, recruitment of Sirt6 near the sites of UV-induced DNA damage restored DNA damage through global genome nucleotide excision repair (NER) (Geng et al., 2020). Also, the binding of Sirt6 to locations of DNA breaks results in SNF2H (a chromatin remodeler) recruitments which protects against genomic instability through chromatin remodeling (Toiber et al., 2013). Besides. Sirt6 promotes chromatin relaxation and DNA damage repair through interacting with Chromodomain helicase DNA-binding protein 4 (CHD4) (Hou et al., 2020). The protective function of Sirt6 is closely associated with the pathogenesis of myriad human diseases. Recently, Wang et al., 2022a reported that vascular smooth muscle cell-specific inhibition of Sirt6 stimulates vascular calcification through repression of DNA damage repair, which proves that Sirt6 also functions as a DNA damage savior in different types of cells other than cardiomyocytes. In addition, knockdown of Sirt6 leads to buildup of DNA damage in KPT-9274-treated acute myeloid leukemia (Zhang et al., 2021). In conclusion, all acquired evidence support the contention that Sirt6 promotes DNA damage repair and regulates various human diseases.

### 3.2 Sirt6 protects against oxidative stress

Oxidative stress happens when the antioxidant defense machinery is overwhelmed by excessive generation of reactive oxygen species (ROS). The ROS family is composed of numerous molecules that wreak havoc in the body. ROS is closely related to pathological changes and catastrophes observed in CVDs, for example, hypertension (Rodrigo et al., 2011) and coronary heart diseases.

The hypothesis that Sirt6 ameliorates oxidative stress-induced damage has been proven by myriad evidence. Sirt6 attenuates oxidative stress-induced damage through different manners such as ROS generation, antioxidant enzymatic activity, exterior stimuli, as well as numerous signaling pathways, respectively.

Reduction of ROS levels by Sirt6 holds the first line of defense against oxidative stress. Loss-and-gain of function experiments supported this notion. For example, it is suggested that Sirt6 overexpression safeguards cardiomyocytes against hypoxia-induced stress through activating AMPK signaling, upregulating Bcl-2 and inhibiting NF-kB, and thus decreasing cellular ROS levels (Maksin-Matveev et al., 2015). It is notable that cardiomyocytes used in the experiments were extracted from Sirt6 overexpression transgenic mice. An *in vitro* model which overexpresses Sirt6 protein in cardiomyocytes is also recommended to confirm previous findings. Similarly, Li et al. reported that Sirt6 decreases ROS levels and alleviates oxidative stress in endothelial cells, therefore Sirt6 could be a nascent target for preserving endothelial cell function and its proper manipulation may exert protective effects on some CVDs (Li PA-O. et al., 2022). Downregulation of Sirt6 expression via transfection of Sirt6 siRNA obviously exaggerates AngII-induced ROS levels in mitochondria (Chen ZA-O. et al., 2022). Samples from tissues of Sirt6-deficient mice showed elevated endogenous hydrogen peroxide levels, which was reversed in cells overexpressing Sirt6. In addition, Sirt6 demonstrated antioxidant activity via enhancing Nrf2 abundance (Liu et al., 2023).

Additionally, it is interesting that Sirt6 also alleviates oxidative stress via manipulating antioxidant enzymatic activities. The antioxidant enzyme system includes the superoxide dismutase (SOD), catalases, glutathione peroxidases (GPXs), and peroxiredoxins (PRXs), etc. (Matés et al., 1999). One paper delineated that cardiac-specific Sirt6 deficient mouse models induces mitochondrial oxidative stress damage during high fat diet treatment via endonuclease G (ENDOG)/superoxide dismutase 2 (SOD2) (Gao et al., 2022).

In the meanwhile, Sirt6 attenuates oxidative stress through various signaling pathways. Myocardial ischemia/reperfusion (I/R) generates oxidative stress-related damage (Zhai et al., 2017). Recent results suggested that upon I/R-induced injury, Sirt6 concentrates antioxidant effects on the NF-E2-related factor 2 (NRF2) signaling pathway (Li et al., 2021). Similarly, it is known that anoxia/reoxygenation (A/R), the *in vitro* models of I/R, also creates a milieu of oxidative stress. Sirt6 plays a protective role in regulating hypoxia/reoxygenation (H/R)-induced cardiomyocyte apoptosis through NF-kB signaling pathway (Chen G. et al., 2022). Besides, recent results showed that Sirt6 protects vascular endothelial cells from AngII-induced oxidative stress through upregulation of Nrf2/ARE signaling cascade, suggesting Sirt6 functions as a promising therapeutic target for the treatment of hypertension related to aberrant endothelial cell function (Yang et al., 2019). Additionally, fatty acid oxidation induces oxidative stress damage, resulting in the dysfunction and apoptosis of pancreatic β-cell, in which Sirt6 co-activates NRF2 and plays an antioxidant role (Tan et al., 2020). Furthermore, some studies revealed that Sirt6 phosphorylation by c-Jun N-terminal kinase (JNK) is necessary for it to physically interacts with poly (ADP-ribose) polymerase1 (PARP1), enhancing DNA DSB repair upon oxidative stress (Li et al., 2021). Moreover, Forkhead box protein O6 (FoxO6) is a novel regulator of oxidative stress and downregulation of FoxO6 upregulates Sirt6 and alleviates cardiomyocyte oxidative stress (Jin AA-O. et al., 2020).

It is hard to translate experimental findings to applicable clinical treatments; however, Zheng et al., 2022 found that Rhaponticum carthamoides (Rha) attenuates myocardial ischemia injury and suppresses oxidative stress in rat myocardial tissue; Rha upregulates Sirt6 levels whereas inhibition of Sirt6 abrogates the positive effect of Rha on ROS levels, suggesting that Rha enjoys a cardioprotective effect on myocardial injury through a Sirt6-dependent signaling pathway. Additionally, accumulation of oxidized low-density lipoprotein (ox-LDL) precipitates atherosclerosis which features oxidative stress. A most recent paper in 2023 reported that Circ_0026218 overexpression dampens oxidative stress damage and restores ox-LDL-induced HUVECs dysfunction via Sirt6 upregulation, implying a protective function of Sirt6 in defending oxidative stress insult (Yang et al., 2023).

In general, these results reiterated the beneficial roles of Sirt6 in oxidative stress-induced damage.

### 3.3 Sirt6 protects against inflammatory response

Our inability to completely deal with inflammatory response is firmly established as essential to the progression and complications of several CVDs. Indeed, the presence of inflammation wreaks havoc in our body, sabotaging tissue integrity as well as functionality (Guo et al., 2022).

Several studies showed that Sirt6 actively functions as a defender against several inflammatory diseases. Latest research demonstrated that circular RNA circ_0026218 ameliorates atherosclerosis development through upregulation of Sirt6 (Yang et al., 2023). Since it is generally accepted that atherosclerosis is characterized as a set of chronic inflammatory responses, Sirt6 plays a significant role in regulating inflammation. Also, the interplay between Sirt6 and pro-inflammatory molecules has been extensively investigated. For example, NF-κB is a mediator which leads to elevated production of pro-inflammatory cytokines such as IL-1β and TNF-α (Zusso et al., 2019). A report held that Sirt6 deacetylation represses the transcriptional activity of NF-κB subunit RelA (Sun et al., 2014). Also, Sirt6 enhances the expression of IκBα, an inhibitor of NF-κB, expediting negative feedback loop signaling that suppresses NF-κB activity (Santos-Barriopedro et al., 2018). To take a step further, Sirt6 also acts downstream of NF-κB. For instance, Sirt6 has been proven to positively speed up the generation as well as releasing of inflammatory cytokine TNF-α at both transcriptional, translational levels and through posttranscriptional modifications. Also, TNF-α is actively synthesized in a Sirt6-dependent signaling pathway in the context of a high intracellular NAD + level, and Sirt6 accelerates mRNA translational efficiency of TNF-α (Van Gool et al., 2009). Taken together, Sirt6 might intervene, to a large extent, in the development of inflammation.

However, effects of Sirt6 on inflammatory response sparks controversies. Although Sirt6 has anti-inflammatory activities through numerous signaling cascades such as histone deacetylation and gene silencing, it is curious that Sirt6 also mounted an active inflammatory response in some specific scenarios. Take TNF-α (a pro-inflammatory cytokine) for example,. An interesting discovery demonstrated that both *in vivo* and *in vitro* acute inhibition of Sirt6 diminish TNF-α secretion and ameliorate LPS-induced septic shock. Another intriguing study reported that short-term genetic deletion of Sirt6 in macrophage samples of obese mice dampens systemic inflammation. Additionally, Sirt6 stabilizes and trans-locates to cytoplasm to promote TNF-α secretion, exaggerating inflammatory response in mice upon LPS stimulation (Bresque et al., 2022). These discoveries seem intriguing and contradict previous findings. It seemed that Sirt6 exhibits a dichotomous effect on inflammation, functioning both as inflammatory response promoter and suppressor. The exact reasons are still unclear; however, one possible explanation held that it all boils down to the fact that different experimental models are employed and the multifaceted effects of Sirt6 on inflammation might be context dependent. Therefore, further inquiries are warranted to interrogate the underlying mechanisms that could explain these different conclusions.

## 4 Sirt6 in cardiovascular diseases and diabetes

It is generally accepted that CVDs are the number one cause of morbidity and mortality among aging-related diseases, claiming more lives than that of cancer and chronic obstructive pulmonary disease combined (Li et al., 2021). Apoptosis, inflammatory response, together with metabolic dysfunction, disrupt normal physiological environment and cause pathological changes in the heart, resulting in the progression of CVDs. Sirt6 enjoys a tremendous effect on these cellular processes and modulates cardiac pathophysiological conditions (Peng and Li, 2020). Here, roles of Sirt6 in atherosclerosis, coronary heart diseases, cardiac I/R injury, hypertension, cardiac hypertrophy, cardiac fibrosis, heart failure, diabetic cardiomyopathy, glucose metabolisms, and DM are reviewed, with emphasis on the molecular network through which Sirt6 regulates myriad CVDs. Much solid evidence now delineates the contention that endothelial remodeling triggers the progression of CVDs, whereas histone modifications are of paramount importance to cardiac remodeling. Since Sirt6 has histone deacetylation activity, it is easily to assume that Sirt6 may actively exert an effect on the pathophysiology of cardiac remodeling, thereby manipulating CVD progression (Yepuri and Ramasamy, 2019).

### 4.1 Atherosclerosis

Coronary artery diseases are the most common types of cardiac diseases associated with Sirt6 activity (D'Onofrio et al., 2018; Zi et al., 2019; Sundaresan et al., 2012). Atherosclerosis has been proven to be the major risk factor for coronary artery diseases such as myocardial infarction and acute coronary syndrome (Nitsa et al., 2018). Atherosclerosis is a chronic progressive inflammatory disease characterized by endothelial cell dysfunction, aggregation of low-density lipoprotein (LDL), presence of scavenger macrophages (foam cells), and sedimentation of cholesterol (Libby, 2021). These components together form atherosclerotic lesions which are also referred to as atherosclerotic plaques. Gradual buildup of atherosclerotic plaques in the arterial inner wall marks the development of atherosclerosis. Once a plaque ruptures, it gives rise to severe cardiovascular events (Matsushima and Sadoshima, 2015), namely, coronary thrombosis and myocardial infarction (Tan et al., 2021). The incidence of atherosclerosis increases with age (Wang and Bennett, 2012).

**FIGURE 2:**
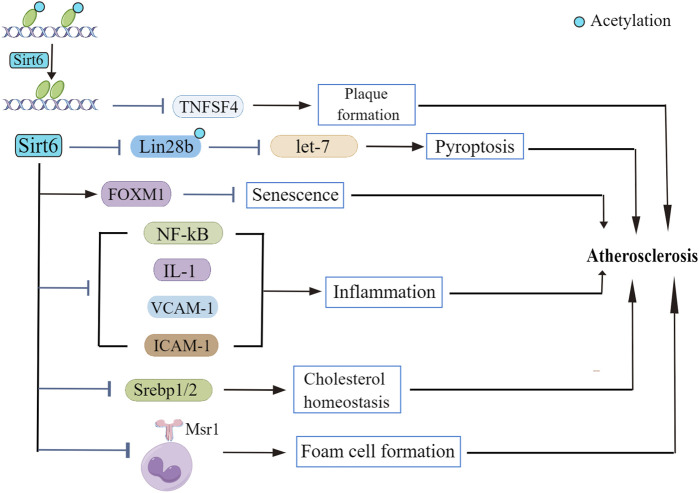
The role of Sirt6 in atherosclerosis. Sirt6 deacetylase activity suppresses TNFSF4 transcription, leading to atherosclerotic plaque formation. Sirt6 modulates Lin28b/let-7 pathway and retards atherosclerosis progression. Sirt6 depletion downregulates FOXM1 expression. Sirt6 exerts regulatory effect on NF-κB as well as its downstream signaling cascades to repress the inflammatory response in atherosclerosis. Sirt6 regulates the transcriptional activity of Srebp1/2, thus improving cholesterol homeostasis. Sirt6 inhibits Msr1 expression and reduces foam cell formation.

Preclinical studies suggested that SIRTs are constantly involved in the regulation of many signaling cascades (Sosnowska et al., 2017). Despite the widely accepted idea that Sirt6 is involved in the maintenance of glucose (Zhong and Mostoslavsky, 2010) and lipid homeostasis (Khan et al., 2021), roles of Sirt6 in the onset and progression of atherosclerosis remains mysterious. Fortunately, evidence explaining participation of Sirt6 in atherosclerotic pathophysiology has been delineated in recent years (Figure 2). Although no study indicated a direct interaction between Sirt6 and atherosclerotic plaque formation (Xu et al., 2016), some recent papers reported that Sirt6 genetic variants have been closely related to the occurrence of plaques in patients (Kane and Sinclair, 2018). Also, according to D'Onofrio et al., 2015, samples from atherosclerotic plaques in diabetic patients show extensive inflammation and presence of oxidative stress, along with a lower Sirt6 expression compared to that of non-diabetic patients. The beneficial roles of Sirt6 in regulating atherosclerotic progress are demonstrated not only in clinical experiments but also in *in vitro* experiments. For example, Xu et al., 2016 found that Sirt6 reduces atherosclerotic lesion formation via amelioration of endothelial dysfunction and vascular inflammation. To the best of our knowledge, several factors accounting for atherogenesis are endothelial cell dysfunction, inflammatory response, and dysregulated lipid metabolism.

Arterial walls that line up with dysfunctional endothelial cells show a propensity for developing atherosclerotic lesions (Gimbrone and García-Cardeña, 2016). Direct evidence showed that SIRT6 inhibition in apolipoprotein E–deficient (ApoE^−/−^) mice demonstrate endothelium-dependent vasodilation dysfunction, plaque size enlargement, as well as more unstable plaque (Liu et al., 2016). In supporting of this, Sirt6 overexpression alleviates minute cholesterol crystal-induced endothelial dysfunction (Jin Z. et al., 2020). Recent studies demonstrated various involvements of Sirt6 in endothelial dysfunction whose occurrence contributes to atherosclerosis progression. Sirt6 influences endothelial function through various signaling pathways. For instance, Sirt6 inhibits mouse vascular endothelial cell pyroptosis via modulation of Lin28b/let-7 pathway in atherosclerosis. Sirt6 downregulates Lin28b expression and demonstrates anti-pyroptosis effect in the inflammatory progress of atherosclerosis (Yao et al., 2022). Recent studies found that isoliquiritigenin (ISL), extracts from traditional Chinese medicine licorice flavonoids, alleviates NLRP3-mediated vascular endothelial cell pyroptosis in HUVECs through Sirt6-dependent signaling cascades, and Sirt6 could be a promising target for ISL in the treatment of atherosclerosis (He et al., 2023). Moreover, Sirt6 binds to proatherogenic tumor necrosis factor superfamily member 4 (*TNFSF4*) gene promoter wherein it catalyzes H3K9 deacetylation, leading to SIRT6-dependent suppression of *TNFSF4* transcription in endothelial cells, demonstrating a positive effect of Sirt6 in atherosclerotic plaque formation (Xu et al., 2016). Besides, one report indicated that silencing of Sirt6 leads to endothelial cell dysfunction by causing DNA damage and telomere malfunction in HUVECs; another claimed that Sirt6 deficiency results in reduced endothelial cell proliferation and cell senescence, thereby crippling normal endothelial functions (Cardus et al., 2013). In addition, Sirt6 depletion lowers forkhead box M1 (FOXM1) expression, leading to endothelial cell senescence (Lee et al., 2020). Moreover, Sirt6 knockdown speeds up cell senescence and activates NF-κB, unveiling a vital role of Sirt6 on inflammation and thus atherosclerosis (Zhang N. et al., 2016). Together, these results indicated that Sirt6 influences endothelial cell function to a large extent and affects atherosclerosis development. To be noted, one paper reported that short-term exposure of endothelial cells to high glucose (HG) downregulates Sirt6 expression (D'Onofrio et al., 2018). Because it is generally accepted that hyperglycemic state is one of the risk factors sensitizing patients to develop atherosclerosis, so it seems plausible that endothelial Sirt6 may influence the regulation of atherosclerosis. However, this study has limitations. HG treatment as an *in vitro* model to simulate atherosclerosis is not flawless. According to the newest study, gel-based cell co-cultures and tissue engineered (TE) are the best available *in vitro* atherosclerosis models developed so far, for they mimic a better microenvironment of atherosclerosis including multiple cell-cell interactions instead of a sole *in vitro* model of hyperglycemic diabetes (Hosseini et al., 2021). Therefore, to better quest Sirt6 implication in atherosclerosis, a more suitable *in vitro* model should be employed in future studies.

Atherosclerosis is also characterized by inflammatory cell infiltration in arterial intima as well as increase in inflammatory cytokine levels. This contention is exemplified by the fact that Sirt6 protects against inflammatory responses in which inflammatory cells play vital roles through regulating NF-kB signaling pathway (Winnik et al., 2015). Sirt6 also acts downstream of NF-κB. Interleukin 1 is a pro-inflammatory cytokine which has an indirect link to atherogenesis (Lappas, 2012; Kuang et al., 2018). Sirt6 depletion increases the expression of interleukin 1, which recruits inflammatory cell (T cells, antigen-presenting cells, and B cells) and are directly associated with atherosclerotic plaque formation (Kuang et al., 2018). In a similar manner, downregulation of endothelial Sirt6 increases vascular cell adhesion molecule1 (VCAM1) as well as intercellular adhesion molecule1 (ICAM1) expression and contributes to the recruitment of inflammatory cells under atherosclerosis condition, resulting in the accumulation of atherosclerotic lesions in the vascular system. It is notable that Sirt6 abrogates the increase in age-induced inflammatory cytokines related to atherosclerosis (Grootaert et al., 2021). More endeavors are needed to excavate information relating to the interplay between Sirt6 and inflammatory cell infiltration as well as changes in inflammatory cytokines.

The theory that Sirt6 prevents atherosclerosis by modulating lipid homeostasis has also been well delineated. Sirt6 has been proven to speed up LDL-C clearance (Kanfi et al., 2010). Along this line, one study claimed that Sirt6 inhibits macrophage scavenger receptor 1 (Msr1) expression, decreasing ox-LDL uptake and foam cell formation (Arsiwala et al., 2020). Also, hepatic Sirt6 prevents hepatic LDL-R degradation and reduces plasma LDL-C levels in mice (Tao et al., 2013). In addition, Sirt6 impedes LDL transcytosis across endothelial cells and hampers atherosclerosis development by deacetylation of caveolin-1 (Zhao et al., 2022). Another study detected a marked decrease in ox-LDL-induced macrophage foam cell formation in lentivirus-infected cell lines overexpressing Sirt6 group compared to that of vector control. Interestingly, it further confirmed that Sirt6 depends on its histone deacetylase activity to avoid atherosclerotic plaque generation via reducing foam cell formation depending on autophagy (He et al., 2017). The latest study concerning the interplay between Sirt6 and lipid homeostasis argued that circular RNA circ_0026218 increases Sirt6 expression and suppresses ox-LDL-induced HUVECs injury and apoptosis, thereby stalling atherosclerosis progression (Yang et al., 2023). Furthermore, our consensus held that hypercholesterolemia triggers atherogenesis by altering arterial endothelial membrane permeability to make lipids easier to access, during which Sirt6 may exert a regulatory effect (Bergheanu et al., 2017). Along this line, Sirt6, which regulates the lipogenic transcription factors (srebp) gene, greatly affects the total cholesterol level in blood circulation. Reports argued that Sirt6 suppresses the transcriptional activity of srebp1/2, and thus improving cholesterol homeostasis (Mostoslavsky et al., 2006). Recently, literature suggested that Sirt6 counters against atherosclerosis through alleviating fatty acid infiltration in the liver in hypercholesterolemic transgenic mice (Mostoslavsky et al., 2006). Tan et al., 2021 found that curcumin promotes cholesterol efflux via miR-125a-5p/Sirt6 axis in macrophages to prevent atherosclerosis Zheng et al., 2021 found that Sirt6/ACE2 signaling pathway prevents cholesterol crystal-induced endothelial dysfunction in HUVECs. Their newest data discovered endothelial Sirt6 overexpression corrects endothelial cell dysfunction (Wang et al., 2023). Sirt6 also influences the modulation of total and LDL cholesterol (Raj et al., 2020), which again corroborates the concept that Sirt6 intervenes in the process of atherogenesis via regulating lipid metabolism. Taken together, results from updated studies shed new light on a possible therapeutic target of Sirt6, which may restore dysregulated lipid homeostasis and hamper atherosclerosis progression.

Interestingly, a peculiar study reported that Sirt6 also plays a deleterious role in atherosclerosis pathogenesis. It demonstrated that Sirt6 causes hemorrhage in atherosclerotic plaques as well as the ability to stabilize hypoxia-induced factor 1α (HIF-1α), which elevates vascular endothelial growth factor (VEGF) expression, accelerates angiogenesis, and increases vessel permeability *in vivo*. This eventually increases the instability and the odds of hemorrhage in unstable atherosclerotic plaques (Yang Z. et al., 2021). Taken together, Sirt6 should be deemed as playing a dichotomous role in modulating the pathophysiology of atherosclerosis. Sirt6 exhibits anti-atherosclerotic effect, and may become possible therapeutic targets for atherosclerosis treatment, together with acute coronary syndrome (Saiyang et al., 2021). However, its pernicious effect also warrants further inquiry, and more experiments are required to inspect the underlying mechanism(s) or the exact molecule(s) by which Sirt6 exerts a regulatory effect on the pathogenesis of atherosclerosis.

### 4.2 Coronary heart disease

Coronary heart disease, which is characterized by atherosclerotic lesion formation and occluded blood flow in coronary arteries, proves to be the most notorious type of cardiovascular diseases that poses a great threat to human health. Once left untreated, it could progress into life-threatening clinical conditions such as acute coronary syndrome or acute myocardial infarction. Therefore, it is imperative to come up with an effective therapeutic approach to tackle this conundrum.

Several studies implied that Sirt6 may function as a positive regulator in the pathophysiology of coronary heart diseases. A recent research paper discovered that serum Sirt6 expression level in patients with stable angina or with acute coronary syndrome was much lower than that of normal patients (Yan et al., 2021). Interestingly, serum Sirt6 levels seems to be lower in patients with acute coronary syndrome than those with stable angina, (but this difference was not statistically significant), reduced serum Sirt6 level accounts for the development of coronary heart diseases such as stable angina and acute coronary syndrome. However, it is well accepted that Sirt6 is intracellularly located, and how does Sirt6 move into the circulation is unclear, and no available studies have addressed this issue. This question requires further inquiry. Nevertheless, this clinical study strongly indicated that Sirt6 exerts a beneficial effect on the pathogenesis of coronary heart disease. This notion was exemplified by another study which showed that Sirt6 is tightly related to coronary artery disease as well as its complications. It reported that the variants of selected Sirt6 genes, namely, rs350844, rs350846, and rs107251, are involved in the susceptibility to coronary artery diseases in a Chinese population (Song et al., 2022). However, this study has limitations. It is restricted to a small sample size and larger sample size taken from various groups are demanded to enrich the results to make it more comprehensive and convincing. Besides, numerous evidence explained that Sirt6 is involved in myocardial infarction. For example, myocardial infarction alters the transcriptional activity of Sirt6 gene promoter and influences Sirt6 protein expression levels (Wang L. et al., 2016). Also, Wang Y. et al., 2022 found that melatonin attenuates myocardial infarction and preserves cardiac function by activating Sirt6-dependent antioxidant pathway. Furthermore, it is reported that metformin therapy mitigates cardiovascular outcomes by improving Sirt6 levels in acute myocardial infarction-prediabetes (AMI-PDM) patients (Sardu et al., 2021). Previous studies connected Sirt6 with myocardial infarction and enriched our current understandings of interactions between Sirt6 and AMI. Moreover, one study showed that a compound xanthenone enjoys cardioprotective effects, namely, antihypertensive effect (Hernández Prada et al., 2008). However, in 2023, a new study argued that xanthenone administration leads to upregulation of Sirt6 via miR-122; Sirt6 upregulation activates ACE2 levels and decreases its substrates angiotensin II, subduing the rampaging storm of inflammatory cytokines and elevated levels of cardiac biomarkers and attenuating myocardial infarction injury (Abdel-Nasser et al., 2023).

### 4.3 Cardiac ischemia/reperfusion injury

Ischemia-reperfusion (I/R) injury usually happens when thrombi, which usually form in the main branch, mostly the left descending artery, or other branches of coronary arteries, result in the occlusion of blood supplement to (part of) the heart, or when the ischemic myocardium receives blood supply after reperfusion treatment. Ischemia causes tissue damage and necrosis, whereas reperfusion, the subsequent remedial attempt, further aggravates this process, leading to increased ROS production as well as apoptosis.

**FIGURE 3:**
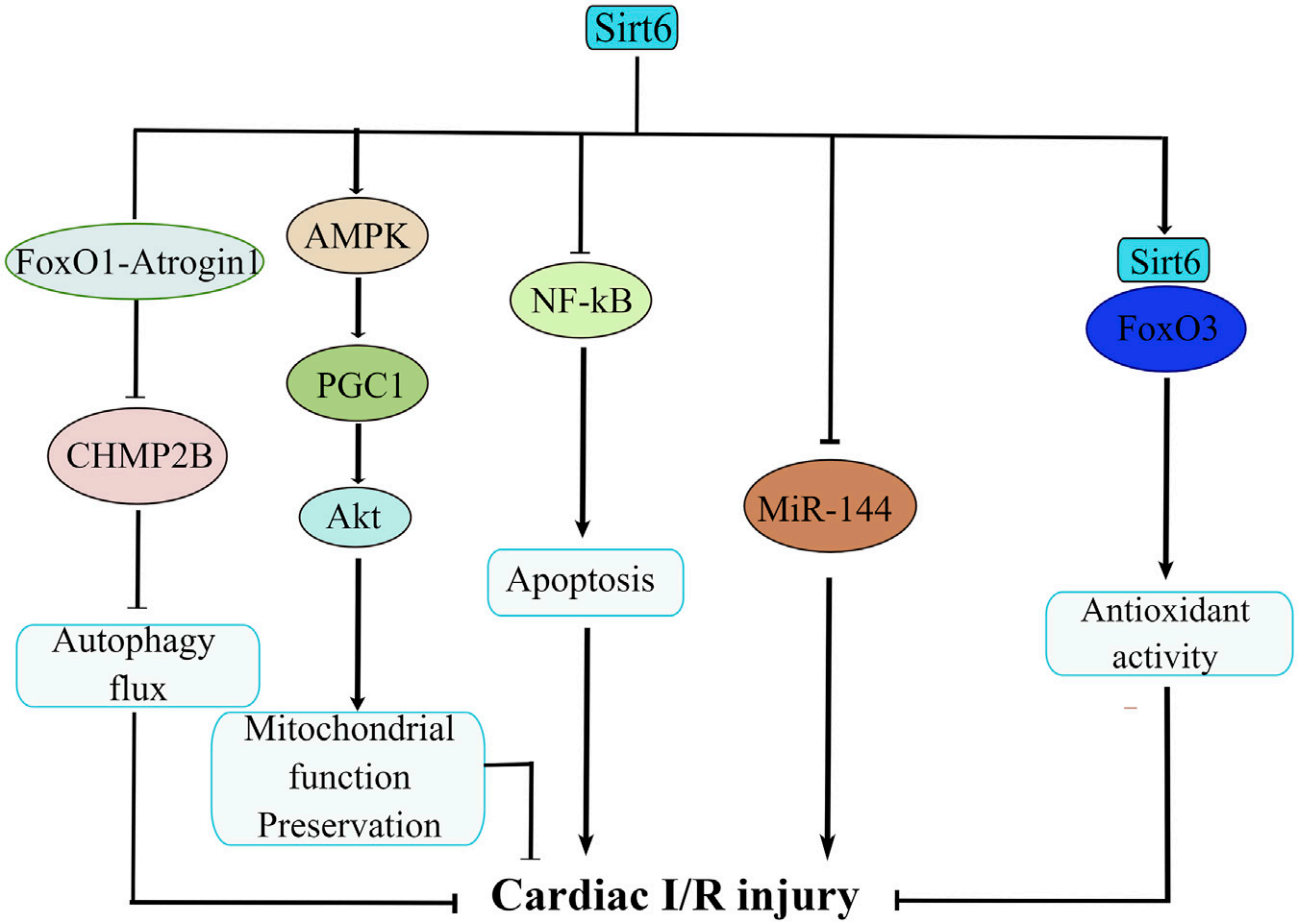
**T**he molecular network of Sirt6 in cardiac ischemia/reperfusion (I/R) injury. Sirt6 activation disassembles CHMP2B accumulation via modulation of FoxO1-Atrogin1 signaling pathway and attenuates cardiac I/R injury. Sirt6 reduces myocardial susceptibility to I/R insult through preserving mitochondrial quality control via SIRT6-AMPK-PGC1α-AKT signaling pathway. Sirt6 inhibits apoptosis via NF-κB signaling. Sirt6 interacts with MiR-144 to reduce H/R-induced myocardial injury. Sirt6/FoxO3 complex upregulates the transcriptional activity of FoxO-dependent antioxidant genes to counteract I/R insult.

Sirt6 is important in the maintenance of cardiac function after I/R-induced injury. The contention that Sirt6 plays a protective role in I/R injury has been well delineated (Figure 3). Regulation of Sirt6 could be viewed as a promising therapeutic approach of cerebral ischemic stroke. For example, studies reported of a beneficial effect of Sirt6 on alleviating cerebral I/R injury by down-regulating MiR-370 (Ruan et al., 2020). In a similar manner, research showed that Sirt6 overexpression attenuates cerebral I/R injury by repressing oxidative stress via activating NRF (Zhang W. et al., 2017). Another study confirmed that Dioscin reduces intestinal I/R injury via Sirt6-mediated pathway (Hu et al., 2018). Besides, sodium sulfide protects brain endothelial cells from stimulated I/R injury via Sirt6-dependent signaling pathway (Hu et al., 2015). In addition, knockdown of Sirt6 generates a larger cerebral infarct size, again revealing a neuro-protective function of Sirt6 (Liberale et al., 2022). Sirt6 not only exerts its beneficial effect on cerebral and gastrointestinal systems, but also on the cardiovascular system. It is reported that the function of Sirt6 in *in vitro* model of cardiac I/R insult has also been examined. For example, some studies confirmed that cardiomyocytes extracted from Sirt6-overexpression mice have less injuries upon hypoxic insult than those from control mice (Cai et al., 2012). Similarly, cardiomyocytes from Sirt6 overexpression transgenic mice exhibits resistance to hypoxia, as well as decreases in ROS production (Matsushima and Sadoshima, 2015). Along this line, when subjected to prolonged hypoxia, cardiomyocytes from Sirt6 overexpression transgenic mice demonstrated improved survival owing to the blockade of necrosis/apoptosis pathways (Maksin-Matveev et al., 2015). The mechanistic workings that pull the trigger behind this also underwent intensive investigation. One paper argued that specific activation of Sirt6 erases charged multivesicular body protein 2B (CHMP2B) accumulation through FoxO1-Atrogin1 signaling pathway and reverses the impaired autophagy flux, thus alleviating myocardial I/R injury in C57BL/6 mice (Li X. et al., 2022). Besides, researchers demonstrated that long-term melatonin treatment mitigates diabetic cardiomyopathy and reduces myocardial susceptibility to I/R insult through preserving mitochondrial quality control via SIRT6-AMPK-PGC1α-AKT signaling pathway (Yu et al., 2021). In supporting of this, myocardial hypoxia/reoxygenation (H/R)-induced injury is significant in mediating the pathogenesis of acute myocardial infarction, and Sirt6 suppresses cell death via NF-kB signaling cascade in myocardial H/R-induced injury (Cheng et al., 2016). Additionally, isoflurane pretreatment markedly regulates miR-744 and its downstream target Sirt6 to alleviate H/R-induced myocardial injury (Chen G. et al., 2022). Moreover, it seemed that Sirt6/FoxO3α signaling pathway also protect cardiomyocyte against I/R injury. Sirt6 forms a complex with FoxO3 in the nucleus, and Sirt6-FoxO3 complex upregulates the transcriptional activity of FoxO-dependent antioxidant genes such as MnSOD to counter against I/R insult (Wang XX. et al., 2016). By now, most cell experiments were conducted focusing on ongoing cellular processes that occurs within cardiomyocytes; however, it is intriguing that silencing of Sirt6 also hampers neutrophil penetration in myocardial infarct area and confines infarct area to a small size during reperfusion (Montecucco et al., 2013). This novel finding is indicative of crucial roles of neutrophil in the attenuation of cardiovascular inflammation (Silvestre-Roig et al., 2020) and offers us insight into indispensable roles of neutrophil in the interplay between Sirt6 and cardiac I/R injury. This finding significantly advances our current knowledge in cardiac I/R-induced injury and inspires us to explore the role of other types of cells in regulating cardiac I/R injury.

Taken together, the interplay between Sirt6 and cardiac I/R injury has been extensively interrogated, but their relationship still requires further inquiry. Until now, pernicious effects of Sirt6 on cardiac I/R insult hasn’t been discovered; however, it is appropriate to prevent its overactivation. Since activation of PARP1 decreases NAD levels, it is significant to contain Sirt6 activation to a suitable level for optimization of its cardioprotective effects.

### 4.4 Hypertension

Hypertension is one of the leading causes of human death around the world and one major risk factors for coronary heart diseases (Kearney et al., 2005). Although many studies have investigated the multifaceted nature of hypertension, the etiology and pathophysiology of primary hypertension remain enigmatic. Endothelial dysfunction is significant for the progression of hypertension as well as its complications (Konukoglu and Uzun, 2017). Thus, determination of Sirt6 as a therapeutic target for attenuating endothelial dysfunction has received major clinical interests.

It is held that Sirt6 corrects endothelial dysfunction and prevents hypertension and its subsequent complications. In supporting of this, literature argued that endothelial Sirt6 plays a regulatory role in preventing hypertension by aiming at GATA5 (GATA binding protein 5) signaling pathway (Guo et al., 2019). Pathologically, hypertension is characterized by arterial calcification, and one paper reported that capsaicin alleviates arterial calcification by upregulating Sirt6-mediated deacetylation of HIF1α (Luo et al., 2022), highlighting a potential therapeutic method for treating arterial calcification, even hypertension. Sirt6 also exerts a modulatory effect on kidney diseases including obstructive nephropathy and chronic kidney disease, contributing to the subsequent outcome of kidney disease-induced hypertension, also known as secondary hypertension (Yang X. et al., 2021). This contention is testified by the fact that Sirt6 expression is reduced in the glomeruli of patients with hypertensive nephropathy, and Sirt6-dependent NRF2-HO1 signaling pathway relieves AngII-induced podocyte apoptosis, which leads to attenuation of hypertensive nephropathy (Fan et al., 2021). Besides, it is widely considered that angiotensin-converting enzyme 2 (ACE2) plays a dominant role in the renin-angiotensin-aldosterone system machinery, hence it may influence the pathogenesis of hypertension. Along this line, Abdel-Nasser et al., 2023 revealed that upregulation of Sirt6 increases ACE2 levels, which possibly alleviates hypertension. Moreover, Sirt6 attenuates hypertensive vascular endothelial injury (Qian et al., 2021). Although many experiments exploring Sirt6 function in secondary hypertension have been conducted, the exact etiology of primary hypertension remains reclusive, to the best of our knowledge. Therefore, attempts to connect Sirt6 with primary hypertension didn’t seem to be easy, and more research are urged to delve into this field.

### 4.5 Cardiac hypertrophy, heart failure, and cardiac fibrosis

In general, hypertrophy is a maladaptive response to physiological and pathological stimuli. Pathological changes of cardiac hypertrophy go through cardiac remodeling, ventricular dilation, weakened myocardial contractility and will, in the end stage, heart failure (Guo et al., 2022). The characteristics of cardiac hypertrophy includes enlarged cardiomyocyte size, interstitial fibrosis, and angiogenesis in heart tissue (Nakamura and Sadoshima, 2018). The incidence of cardiac hypertrophy keeps increasing as people ages (Lakatta and Levy, 2003).

Sirt6 has long been hailed as a longevity protein. Recent studies have demonstrated regulatory effects of Sirt6 on cardiac hypertrophy (Matsushima and Sadoshima, 2015). This was first indicated by evidence showing that decreased Sirt6 protein expression levels and activities were detected in heart samples from both mouse and human failing hearts (Kugel and Mostoslavsky, 2014). Then, literature is indicative of regulatory roles of Sirt6 in cardiac hypertrophy using loss-and-gain of function mouse models. It argued that Sirt6 knockout mice exhibits shortened lifespan and can’t survive long enough for subsequent experiments. Therefore, Sirt6 heterozygous mouse models showing 50% reduced Sirt6 protein expression were employed in this study. Such Sirt6 half-depletion mouse models developed cardiac hypertrophy, whereas mouse models overexpressing Sirt6 displayed significant attenuation of cardiac hypertrophy (Matsushima and Sadoshima, 2015). A similar study further attested to this contention. It claimed that cardiac-specific deletion of Sirt6 notably thickens ventricular walls, increases cardiac interstitial fibrosis, and exacerbates cardiac hypertrophy; whereas Sirt6 overexpression mitigates pressure-overload-induced cardiac hypertrophy (Guo et al., 2022). However, the *in vivo* model of Sirt6 knockout mouse has limitations because shortened lifespan impedes in-depth experiments whereas Sirt6 heterozygous mouse models is not effective enough because Sirt6 expression level is just half-depleted. Therefore, efforts are mandated to construct a better animal model for future study. Researchers also revealed the cardioprotective effect of Sirt6 in a mouse model of Transverse Aortic Constriction (TAC)-induced heart failure. They claimed that Sirt6 overexpression ameliorates impairment of cardiovascular function, decreases TAC-induced myocardial inflammation, reduces infarct size, and improves the survival of TAC mice (Li et al., 2017). These findings directly confirmed that decreased Sirt6 expression level is an indicator for developing cardiac hypertrophy.

**FIGURE 4:**
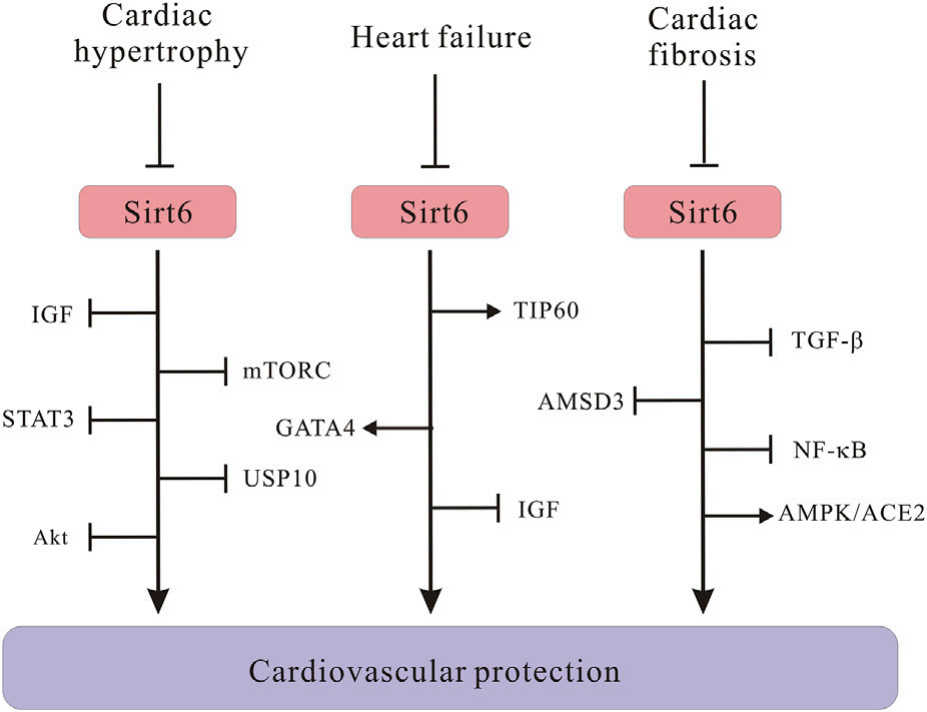
Schematic illustrating Sirt6 signaling molecules in the development of cardiac hypertrophy, heart failure and cardiac fibrosis. Sirt6 downregulation activates IGF/Akt signaling pathway, leading to cardiac hypertrophy. Sirt6 negatively regulates mTORC and STAT3 signaling to protect against cardiac hypertrophy. Sirt6 interacts with USP10 in the pathogenesis of cardiac hypertrophy. The Sirt6/TIP60/GATA4 axis ameliorates doxorubicin-induced cardiotoxicity leading to heart failure via anti-apoptotic signaling pathways. Sirt6 inhibits IGF signaling and prevents cardiac hypertrophy from progressing into heart failure. Sirt6 downregulates TGF-β/AMSD3 axis and NF-κB. Sirt6 positively regulates AMPK/ACE2 axis to alleviate cardiac fibrosis.

To take a step further, molecular mechanisms through which Sirt6 regulates cardiac hypertrophy have also been intensively explored (Figure 4). Many researchers argued that Sirt6 is a negative modulator in the progression of cardiac hypertrophy and heart failure. The progression of cardiac hypertrophy is also associated with dysregulated IGF signaling, intracellular NAD levels, activation of autophagy, as well as upregulation of signal transducer and activator of transcription 3 (STAT3) (Sundaresan et al., 2012; Liu et al., 2014; Zhang X. et al., 2016; Lu et al., 2016). Under pathological stress, reduced Sirt6 expression undermines H3K9 deacetylation, which in turn promotes binding of transcription factor c-Jun; the activation of downstream IGF-Akt signaling pathway results in cardiac hypertrophy (Sundaresan et al., 2012). This was further confirmed by another paper which declared that Sirt6 directly inhibits IGF signaling cascade, slowing down the pathogenesis of cardiac hypertrophy (Lu et al., 2016). Besides, increasing NAD + level in rat cardiomyocytes through overexpression of nicotinamine mononucleotide adenylyltransferease (NMNAT) safeguards against cardiac hypertrophy via Sirt6-mediated pathway, suggesting that NMNAT2 overexpression would be unable to stop AngII-induced cardiac hypertrophy without Sirt6 intervention. Similarly, a new PARP-1 inhibitor, AG-690/11026014 (6014), has been revealed to retard AngII-induced cardiac hypertrophy, partly because of the activation of Sirt6 by elevating intracellular NAD + levels (Liu et al., 2014). Interestingly, on the contrary, overexpression of Sirt6 in rat cardiomyocytes significantly dampens hypertrophic responses, which again manifested that Sirt6 plays crucial roles in regulating cardiac hypertrophy pathogenesis (Cai et al., 2012). In addition, in cardiomyocytes, autophagy primarily recycles and cleanses damaged or dysfunctional cellular organelles to maintain cellular homeostasis, however, excessive activation of autophagy cripples cell function and results in heart failure (De Meyer and Martinet, 2009). Lu et al. showed that Sirt6 ameliorates cardiac hypertrophy through FoxO3/autophagy-dependent signaling pathway upon isoproterenol stimulation. Sirt6 inhibits Akt activation, thus, promoting FoxO3 activation and augmenting autophagy for clearing aberrant and misfold proteins (Lu et al., 2016). Moreover, mTORC1, which is a serine/threonine protein kinase, regulates autophagy process. An *in vitro* experiment proved that Sirt6 represses mTOR-related gene expression and protein biosynthesis, thus, ameliorating cardiomyocyte enlargement (Ravi et al., 2019), connecting Sirt6 with autophagic process via mTORC signals. However, it is notable that most of these experiments which are indicative of positive roles of Sirt6 in cardiac hypertrophy were conducted using primary rat cardiomyocytes, whether Sirt6 exerts a similar effect on other types of cell models deserves further inquiry. Elsewhere, researchers argued that STAT3 activation also controls cardiac remodeling and cardiac failure. Sirt6 upregulation silences STAT3 activation induced by phenylephrine (PE) and ameliorates cardiomyocyte hypertrophy (Zhang X. et al., 2016). Besides, Sirt6, depending on its deacetylase activity, represses the expression of nuclear factor of activated T cells c4 (NFATc4) and protects against cardiac hypertrophy (Li et al., 2019). Additionally, a study reported of important roles of Sirt6 in regulating in cardiac hypertrophy associated with deubiquitinase ubiquitin-specific protease 10 (USP10) (Liu et al., 2022). The USP10 attenuates aortic constriction-induced cardiac hypertrophy by directly regulating Sirt6 levels (Zhang DH. et al., 2020). To be noted, it is crucial to translate experimental findings to feasible clinical therapeutic approaches. Up until now, nobody has identified a deleterious role of Sirt6 regarding cardiac hypertrophy. More research efforts are needed to deeply investigate regulatory effects of Sirt6 in cardiac hypertrophy.

Dysregulated energy metabolism in cardiomyocyte is recognized as a significant pathogenic mechanism accounting for heart failure (Lopaschuk et al., 2021). For example, Sirt6 inhibition results in congregation of lipid droplets in cardiomyocytes, whereas overexpression of Sirt6 increases fatty acid transporter expression levels both *in vivo* and *in vitro*. Taken together, Sirt6 alleviates lipid accumulation in cardiomyocytes and cardiac dysfunction through modulating lipid uptake (Khan et al., 2021). In addition to energy metabolism, Sirt6 manages to preserve cardiac function through other manners. For example, the Sirt6/TIP60/GATA4 axis connects gene transcription with epigenetic activation, ameliorating doxorubicin-induced cardiotoxicity via anti-apoptotic signaling pathways (Peng et al., 2020). Other results indicated that Sirt6 blocks IGF signaling by interacting with c-JUN, inhibition of c-JUN or IGF signaling retards cardiac hypertrophy of Sirt6-deficient mouse hearts, thus preventing cardiac hypertrophy from progressing into heart failure (Xiao et al., 2012). Moreover, literature demonstrated that Sirt6 also maintains normal cardiac function and attenuates heart failure in mouse model of diabetes (Wu et al., 2022).

Myocardial fibrosis, the expansion of cardiac interstitium through deposition of extracellular matrix proteins, is a common pathophysiologic companion of many different myocardial disorders. Cardiac fibrosis, which features the reduction of fibroblasts and increase of myofibroblasts, accelerates the pathological process of cardiac remodeling (Frangogiannis, 2021). According to literature, the deacetylating activity of Sirt6 decreases TGF-β/AMSD3 signaling pathway gene expression, which means Sirt6 is a possible candidate for regulating TGF-β signaling to diminish fibrosis (Maity et al., 2020). Also, in accordance with other CVDs, Sirt6 also regulates cardiac fibrosis via nuclear factor κB (NF-κB) signals. Sirt6 has been reported to repress NF-κB activation, retarding cardiac fibroblasts differentiation into myofibroblasts and suppressing cardiac fibrosis (Maity et al., 2020). On the contrary, depletion of Sirt6 enhances transcriptional activity of NF-κB and exacerbates cardiac fibroblasts differentiation into myofibroblasts, unraveling a novel finding of Sirt6 as a crucial role in cardiac fibrosis (Tian et al., 2015). Another discovery confirmed that Sirt6 has cardioprotective effect on cardiac fibrosis through upregulation of AMPK-ACE2 signaling pathway, implying that manipulating Sirt6 has promising therapeutic importance for cardiac fibrosis and cardiac diseases (Zhang ZZ. et al., 2017). A latest study published in 2023 discovered that hepatic-specific depletion of Sirt6 in mice has been demonstrated to exhibit hepatic fibrosis (Dong, 2023), although one limitation of this experiment is that it was conducted in *in vivo* models of hepatic fibrosis instead of cardiac fibrosis, it arose researchers’ interests in the regulatory function of Sirt6 in cardiac fibrosis and enlightened more people to devote their time into this direction.

### 4.6 Glucose metabolism, diabetes and diabetic cardiomyopathy

The incidence of diabetes is increasing rapidly worldwide. As our knowledge expands, extensive research has been conducted in exploring potential therapeutic manners in the treatment of diabetes. Diabetes mellitus is a chronic metabolic disorder featuring high blood glucose level and insulin resistance, and it gives rise to numerous cardiovascular and metabolic complications including hyperlipidemia and coronary heart diseases, if not properly handled (Raj et al., 2020).

Blood glucose level should be kept at a specific point to provide enough energy for organ and tissue. Perturbations in glucose homeostasis could account for the progression of diabetes and other metabolic disorders (Zhong et al., 2010). The fundamental role of Sirt6 in regulating glucose metabolism was firstly identified by evidence which reports of serious hypoglycemia in Sirt6-deficient mice, in accordance with increased glucose uptake from muscle and adipose tissue (Zhong and Mostoslavsky, 2010). Both *in vivo* and *in vitro* evidence showed that increased glucose uptake may be due to deficiency of Sirt6 (Zhong and Mostoslavsky, 2010; Cencioni et al., 2015).

Some people held that Sirt6 regulates glucose metabolism and diabetes mainly by targeting insulin resistance and pancreatic β cell function. For example, reports suggested that Sirt6 exerts its regulatory effects on pancreatic β cell function, which is essential for keeping blood glucose level at a specific point (Demir et al., 2017). In addition, β cell-specific Sirt6-deficient mice exhibits increased sensitivity and susceptibility to streptozotocin (STZ)-induced β cell apoptosis, impairing pancreatic function and thus, blood glucose level (Qin et al., 2018). Another study claimed that Sirt6 activation protects against insulin resistance in neonatal rat cardiomyocytes in H9C2 cell line (Guo et al., 2019). Furthermore, muscle specific Sirt6 inhibition reduces AMPK activity and disrupts glucose homeostasis and insulin sensitivity. Additionally, myeloid-specific knockout of Sirt6 mice on a high-fat diet develops insulin resistance, a great liability for diabetes (Cui et al., 2017; Lee et al., 2017). In an analogous manner, hepatic-specific ablation of Sirt6 in mice increases liver steatosis while neural-specific deletion of Sirt6 in mice promotes diet-induced obesity and insulin resistance (Kanwal et al., 2019).

Elsewhere, Sirt6 also affects gluconeogenesis. Along this line, Sirt6 deacetylases acetyltransferase GCN5, increasing proliferator-activated receptor γ coactivator-1α (PGC-1α) activation, and thus, manipulating gluconeogenesis (Dominy et al., 2012). Taken together, it seemed that Sirt6 influences many aspects of the etiology of diabetes.

The underlying mechanism through which Sirt6 modulates diabetes and glucose metabolism also underwent intensive investigations. Sirt6 downregulates gluconeogenesis in liver through FoxO1-mediated signaling cascade, thus protecting a diabetic mouse model from hyperglycemia. Dominy et al., 2012 demonstrated that Sirt6 knockout decreases GCN5 activity, reducing the acetylation level of PGC-1α, which controls the expression of gluconeogenic genes. Interestingly, Balestrieri et al. discovered that samples from atherosclerotic lesions of DM patients exhibits lower Sirt6 protein expression level compared with from atherosclerotic lesions of non-diabetic patients, showing that Sirt6 intervenes in the pathogenesis of atherosclerosis patients with diabetes mellitus; this makes us reminiscent of the fact that diabetes exaggerates the clinical outcome of myocardial I/R injury (Rui et al., 2012), and future study should focus on roles of Sirt6 in coronary heart diseases patients with diabetes mellitus. The author further suggested that decreased Sirt6 level is associated with increased oxidative stress (Pan et al., 2016). In conclusion, these results indicated that Sirt6 exerts its beneficial leverage on diabetes by numerous signaling pathways.

Diabetic patients are predisposed to develop a specific cardiomyopathy which is often referred to as diabetic cardiomyopathy. Diabetic cardiomyopathy is a pathophysiological condition induced by diabetes mellitus and could progress into heart failure. Pathologically, diabetic cardiomyopathy demonstrates both structural and functional impairment of diabetic myocardium that sometimes occurs without complications such as coronary heart diseases (Sosnowska et al., 2017). It is generally accepted that DM and insulin resistance contribute to the initiation and progression of diabetic cardiomyopathy (Tan et al., 2020). Long-term exposure of cardiomyocytes to the diabetic milieu of hyperglycemia and elevated fatty acids levels lays foundation for the initiation of diabetic cardiomyopathy. It is also known that Sirt6 regulates glucose metabolism and lipid metabolism (Zhong and Mostoslavsky, 2010; Khan et al., 2021). Therefore, it is possible that Sirt6 impacts diabetic cardiomyopathy pathophysiology. Impairment of Sirt6 function worsens pathological cellular processes related to the progression of diabetic cardiomyopathy. Loss-and-gain of function experiments have testified this contention. For example, a paper claimed that Sirt6 inhibition exacerbates diabetic cardiomyopathy by intensifying oxidative stress and inflammation in H9c2 cell line (Huang et al., 2021) whereas Sirt6 overexpression prevents the heart from developing obesity-mediated diabetic cardiomyopathy (Kanwal et al., 2019). Most recently, literature reported that melatonin-involved activation of Sirt6-related signaling pathway mitigates diabetic cardiomyopathy (Yu et al., 2021). Another paper held that Sirt6-KO cardiomyocyte endothelial cells (CMECs) were discovered to exacerbate diabetic cardiomyopathy, as indicated by aggravated perivascular fibrosis, cardiomyocyte hypertrophy, and decreased cardiac function. These findings suggested Sirt6 as a potential therapeutic strategy for dealing with diabetic cardiomyopathy (Zhang YA-O. et al., 2020). However, due to the scarcity of evidence, more research endeavors are encouraged in this direction.

## 5 In summary

The biological functions and fundamental roles of Sirt6 in regulating CVDs and DM have been well described. In terms of biological function, Sirt6 promotes DNA damage repair, regulates oxidative stress, and attenuates inflammatory responses. Since crippled biological functions of Sirt6 contribute to CVDs, it is valuable for us to study them in a broader spectrum. The important biological effects that Sirt6 exerted on CVDs and DM influence our health and well-beings. Informed of roles of Sirt6 in mediating variegated mechanisms in various diseases, targeting Sirt6 and its downstream mechanistic cascades could be a promising therapeutic approach for the treatment of CVDs as well as its complications and other abnormalities. Although the discoveries imposed by hyperactivation of Sirt6 in human studies are not fully understood, overexpression of Sirt6 may be a promising therapeutic approach (Yepuri and Ramasamy, 2019). However, translation of experimental findings to effective clinical therapeutic approaches has been unsuccessful by now. After all, it comes down to the fact that molecular mechanisms by which CVDs and DM work are not fully understood. There is no denying that more endeavors are required to further our understanding of the underlying mechanism(s) through which Sirt6 regulates CVDs and DM (Sociali et al., 2017). In addition, on the contrary to protective roles of Sirt6 in CVDs, it is intriguing that some studies demonstrated deleterious effects of Sirt6. Therefore, deeper studies that explained these different conclusions are also mandated.
